# Acute kidney injury biomarkers offer the opportunity to reduce exposure to nephrotoxic drugs

**DOI:** 10.1186/cc14375

**Published:** 2015-03-16

**Authors:** M Ostermann, L Forni, K Kashani, M Joannidis, A Shaw, M Chawla, JA Kellum, on Behalf of Sapphire Investigators

**Affiliations:** 1 Guy's & St Thomas Foundation Hospital, London, UK; 2 Royal Surrey County Hospital, Guilford, UK; 3 Mayo Clinic, Rochester, MN, USA; 4 University Hospital Innsbruck, Austria; 5 Vanderbilt University Hospital, Nashville, TN, USA; 6 George Washington University Medical Center, Washington, DC, USA; 7 University of Pittsburgh, PA, USA

## Introduction

Tissue inhibitor of metalloproteinase-2 (TIMP-2) and insulin-like growth factor binding protein 7 (IGFBP7) are specific urinary biomarkers which can predict acute kidney injury (AKI) in critically ill patients within 12 hours [1]. A [TIMP-2]·[IGFBP7] result >0.3 (ng/ml)^2^/1,000 is associated with seven times the risk of AKI compared with a test result ≤0.3 [2]. The aim of our study was to explore the use of potentially nephrotoxic medications within the window between a positive biomarker test and the diagnosis of AKI stage 2 or 3.

## Methods

We identified all patients enrolled into the Sapphire study [1] who received at least one potentially nephrotoxic drug on the day of AKI (defined by stage 2 or 3 as per KDIGO classification). We subsequently determined the proportion of patients who had a [TIMP-2]·[IGFBP7] result >0.3 (ng/ml)^2^/1,000 before meeting the criteria for AKI.

## Results

Of 184 patients who developed AKI, 58% received one or more potentially nephrotoxic drug on the day of AKI. Eighty-nine percent of these patients had a positive biomarker test ≥12 hours earlier. In 41% of patients receiving one or more nephrotoxic drug on the day of AKI, at least one nephrotoxic medication was stopped within 1 day of AKI, and in 24% of patients all nephrotoxic drugs were stopped within 1 day of AKI, which implies that these medications were not absolutely necessary.

## Conclusion

Nephrotoxic medications are commonly used in patients who develop moderate or severe AKI. The [TIMP-2]·[IGFBP7] test could have identified many of these patients earlier and would have offered an opportunity to reduce exposure to non-essential nephrotoxic drugs.
